# Olfactory Capacity and Obesity in Chilean Adolescents †

**DOI:** 10.3390/nu17243903

**Published:** 2025-12-13

**Authors:** Samuel Duran Agüero, Gary Goldfield, Karina Oyarce, Camila Riquelme, Julia Pozo, Ana María Obregón-Rivas

**Affiliations:** 1 Escuela de Nutrición y Dietética, Facultad de Ciencias de la Rehabilitación y Calidad de Vida, Universidad San Sebastián, Santiago 7510000, Chile; 2 Children’s Hospital of Eastern Ontario Research Institute, Ottawa, ON K1H 8L1, Canada; 3 Laboratorio de Neuroinmunologia, Departamento de Bioquímica Clínica e Inmunologia, Facultad de Farmacia, Universidad de Concepción, Concepción 4070386, Chile; 4 Escuela de Medicina, Facultad de Medicina y Ciencia, Universidad San Sebastián, Concepción 4080871, Chile; 5 Escuela de Nutrición y Dietética, Facultad de Ciencias de la Rehabilitación y Calidad de Vida, Universidad San Sebastián, Concepción 4080871, Chile

**Keywords:** olfactory capacity, eating behavior, nutrition status

## Abstract

Background: Childhood obesity is a global issue, influenced by energy-dense foods and powerful cues that affect brain areas regulating food intake. The olfactory system, linked to food preferences and consumption, is inversely related to body mass index. However, no studies have assessed the possible effect of eating behavior traits on the relationship between olfactory capacity and obesity. Objectives: The aim of this study was to examine whether olfactory capacity, eating behavior traits, and body mass index are associated with obesity in adolescents. Methods: An analysis of 204 Chilean adolescents was undertaken in a cross-sectional study. The proportion of participants with normal weight was found to be 39.2%, that of overweight was 25.9%, and that of obesity was 34.8%. Anthropometric measurements (weight, height, BMI Z-score), eating behavior, and olfactory capacity were evaluated. The Child Eating Behavior Questionnaire (CEBQ) and Food Reinforcement Value Questionnaire (FRVQ) were used to assess eating behavior. The Sniffing sticks test was used to assess olfactory capacity. Results: In the global sample, 1.0% had anosmia, 20.5% had hyposmia, 61.0% had normosmia, and 17.5% were supersmellers. Girls showed higher odor identification percentages than boys (*p* = 0.01). No gender differences were found in olfactory threshold, discrimination, identification, or TDI (threshold–discrimination–identification) scores, nor nutritional status. Stratified analysis revealed that girls with obesity had significantly lower odor discrimination capacity compared to those with normal weight. Conclusions: the study highlights a potential link between olfactory function and obesity, with obese girls showing reduced odor discrimination compared to normal-weight girls. Further research is needed to explore these mechanisms and their implications for targeted obesity interventions.

## 1. Introduction

Obesity is a rapidly growing pandemic with the important feature of being preventable [1]. While this phenomenon remains one of the most serious public health issues in developed countries, its prevalence is also increasing rapidly in many developing countries [2]. Increasing availability of energy-dense, palatable foods overrides homeostatic satiety and influences the reward and emotional centers of the cortico-limbic system [3]. In this regard, the olfactory system is one of the most important. Humans require smell for the perception of flavor [4], and it contributes to their food selections and eating patterns by contributing in the hedonic evaluation of food [5].

The olfactory function can affect energy balance and body weight, as well as varying metabolism- and feeding-related behaviors. Changes in olfactory function can be manifested as increases or decreases in olfactory identification, detection threshold, discrimination, or hedonic value. Evidence proposes that the olfactory system is closely related to neural systems that regulate food intake by sensing metabolic state [6]. Increasing research on the gut–brain axis and gut microbiota further highlights the complexity of these connections in metabolic regulation [7]. Additionally, genes and epigenetic differences also impact how smells are perceived and influence eating behavior and metabolism [8].

Pastor et al. (2016) showed that participants living with obesity have a lower olfactory ability than normal-weight subjects, and olfactory capacity was inversely correlated with age, BMI, % body fat, and fasting plasma concentrations of the endocannabinoid arachidonoyl glycerol 2-AG (an endocannabinoid that increases appetite) [9]. In this context, Skrandies et al., 2015, showed that an increasing BMI in healthy adults was associated with decreased olfactory and taste sensitivity and that body weight influences gustatory and olfactory perception [10]. Recently, a systematic review developed by Peng et al. (2019) found solid evidence that individual body weight is negatively correlated with olfactory ability [2].

Among children and adolescents, few studies examine olfaction capacity and obesity. Only one published study has been conducted on this topic, by Obrebowski et al. (2000) [11]. In the study, children with obesity had significantly lowered thresholds for detecting and identifying odors, as well as for tasting odors (this means that they are more sensitive to odors; they can detect and identify smells at much lower concentrations than their normal-weight peers). Also, high reactivity to olfactory food cues was observed in overweight children [11]. Recently, a study revealed that OBV (olfactory bulb volume) is higher in children with obesity, and a positive correlation between OBV and BMI was found in overweight and obese children. Nevertheless, in the group of those living with severe obesity, a negative correlation was observed [12].

Current knowledge of olfactory processing is largely derived from rodent models. Odor-evoked responses are thought to originate from nasal mucosal first-order neurons leading to the olfactory bulb. As a result, information is relayed to the anterior olfactory nucleus, the piriform cortex, and the amygdala, which together make up the primary olfactory cortex [13].

The theory suggests that the relationship between olfaction and obesity is mediated by complex hormonal and metabolic interactions [6]. Fasting states enhance olfactory sensitivity through increased levels of orexigenic hormones such as ghrelin and orexins, whereas satiety and obesity are associated with elevated anorexigenic signals, including leptin and insulin, that exert inhibitory effects on olfactory processing [14].

In individuals with obesity, typical alterations such as hyperglycemia, insulin resistance, and hyperleptinemia may directly impact both peripheral and central olfactory pathways [15]. Additionally, adipokines and gut-derived hormones can modulate odor perception and hedonic evaluation, thereby influencing eating behavior. Animal studies further indicate that obesity-related olfactory dysfunction can persist even after weight loss, suggesting long-lasting structural changes within the olfactory system [2,16].

As the sense of smell impacts food selection and food consumption, it also contributes to body weight and BMI regulation. We investigated the association between olfactory capacity, body mass index, and body composition in Chilean adolescents aged 10-16 years. Based on the literature in adults, we hypothesized that there would be an inverse association between olfactory capacity and body mass index in adolescents. Also, we hypothesized that olfactory status (normosmic or hyposmic) influences BMI and eating behavior traits, where being hyposmic would be associated with higher BMI and unhealthy eating traits.

## 2. Materials and Methods

We conducted a cross-sectional study involving 80 normal-weight (≤85th percentile), 53 overweight (≥85th–<95th percentile), and 71 obese (≥95th percentile) Chilean adolescents (10–16 years old, both genders) from Chile, classified according to the WHO International criteria, from December 2022 to May 2024. A non-probabilistic sampling method was employed. Participants were primarily recruited from schools in Concepción between April 2023 and December 2025 through written invitations, community open calls, and virtual or social media platforms (www.uss.cl, accessed on 1 April 2023), as well as telephone calls. Adolescents and their parents were invited to San Sebastián University to learn about the study, where adolescents provided authorization and parents signed an informed consent form approved by the Ethics Committee (#19-23). The study adhered to the Singapore Statement. The study’s inclusion criteria were adolescents aged 10–16 years with nutritional status categorized as normal weight (≤p85), overweight (≥p85–<p95), or obese (≥p95) based on the WHO International criteria. Exclusion criteria included (a) known anatomical or functional nasal issues, (b) participation in a weight reduction program, (c) use of medications affecting body weight, (d) undergoing weight loss treatment, (e) a history of chronic medical conditions, (f) genetic syndromes, (g) a diagnosis of eating disorders such as anorexia, bulimia, or binge eating, and (h) having a cold or having had a cold in the past two weeks.

A total of 204 adolescents were recruited, and the following sociodemographic and nutritional variables were collected: sex (male/female), age (years), weight and height, BMI, z-score for BMI, waist circumference, % of body fat, olfactory variables, and eating behavior.

*Anthropometry:* Weight, height, and waist circumference were measured using a weight scale, with a stadiometer included (Seca 700, Seca GmbH & Co. KG, Hamburg, Germany; 100 g and 0.5 cm sensitivity, respectively). Each subject was measured at room temperature, with underwear on, without shoes, their body weight distributed uniformly on both feet, their back to the stadiometer, with the heels and knees together, a straight back, and arms relaxed at the side. To measure height, each participant stood with their heels together, arms at the sides, legs straight, and shoulders relaxed. This measurement was performed after a deep inhalation with the head in the Frankfort Horizontal Plane position [17]. BMI (body mass index) was estimated as the quotient of weight (in kilograms) divided by the square of height in meters (kg/m^2^). Adolescents exceeding the 95th BMI percentile (WHO standard curve 2007) [18] (https://www.who.int/tools/growth-reference-data-for-5to19-years, accessed on 7 December 2025) were considered obese. The percentiles and Z-scores of height, weight, and BMI were calculated using WHO AnthroPlus. In addition, body composition was assessed at 0900 after an overnight fast by leg-to-leg bioelectrical impedance according to the manufacturer’s guidelines with a Tanita TBF-300 MA (Tanita Corporation, Tokyo, Japan). The pubertal stage (development of breasts, genitals, and pubic hair) was self-documented in all adolescents according to the Tanner classification.

Waist circumference was measured using a non-elastic tape midway (Seca 201, Seca GmbH & Co. KG, Hamburg, Germany) between the lowest border of rib cage and the upper border of iliac crest, at the end of normal expiration. Hip circumference was measured at the widest part of the hip at the level of the greater trochanter. All measurements were in centimeters (cm) and rounded to the nearest millimeter. Body composition was assessed at 09:00 after an overnight fast, by leg-to-leg bioelectrical impedance according to the manufacturer’s guidelines with a Tanita TBF-300 MA (Tanita Corporation, Tokyo, Japan).

### Olfactory Capacity Tests

This assessment of olfactory performance was conducted using a test known as “Sniffin Sticks” (Burghart Messtechnik GmBH, Wedel, Germany), which uses pen-like devices to disperse odors over a short period of time. This test was previously described and validated [19,20,21] and is considered appropriate for routine clinical assessment of olfactory performance. This test is made up of three sub-tests: (a) odor threshold (OT); (b) odor discrimination (OD); and (c) odor identification (OI). OT is defined as the lowest concentration of a particular odor compound perceivable by the human sense of smell. OD and OI show the capacity to differentiate and recognize odorants, respectively. OD and OI are related to cognitive aspects of olfaction, while OT is more sensorial [22]. OT is evaluated with n-butanol, while OD and OI are assessed with sixteen common odors: banana, garlic, peppermint, orange, leather, cinnamon, lemon, rose, coffee, apple, clove, pineapple, aniseed, and fish [20]. Participants were assessed individually in a well-ventilated room with eye masks on and were requested not to smoke, chew gum, or eat any products during the previous hour to the start of the test. The tests was performed between breakfast and lunch. A trained nutritionist carry out the tests in the following order: OT, OD, and OI. (a) Olfactory threshold (OT) test: Using a triple-forced choice paradigm, detection thresholds were measured by employing a single staircase method. Three pens were presented in a randomized order; two pens contained odorless samples, and the third an odorant sample at a particular dilution. A total of 16 odor concentrations were tested. The task of the subject was to indicate which pen contains the odorant. The odorant concentration was increased if the subject chose an odorless pen and reduced if the correct pen was recognized twice, triggering a reversal of the staircase. The mean of the last four staircase reversal points of a total of seven reversals was used as the estimated threshold. The range of this score was from 0 to 16 points. The higher the score, the higher the olfactory threshold capacity. (b) Olfactory discrimination (OD) test: The adolescents were asked to discriminate between 16 triplets of odors. In each group, two odors were equal and one odor different. The task was to recognize the pen in which the odor was different. The total score was calculated as the sum of correct responses, ranging from 0 to 16 points. The higher the score, the higher the olfactory discrimination capacity. (c) Olfactory identification (OI) test: A pen with an odor was presented to the adolescents, and they were asked to identify the odor by choosing it on a card from a list of four descriptors, only one of which would correctly identify the odor. For this purpose, the eye mask was removed (only for reading the card). The total score was calculated as the sum of correct responses, ranging from 0 to 16 points. The higher the score, the higher the olfactory identification capacity.

Threshold–discrimination–identification score (TDI): This was calculated as the sum of scores from the three subtests’ results, giving the olfactory threshold–discrimination–identification (TDI) score. This final score could range from 0 to 48 points. The higher the score, the higher the olfactory capacity. TDI declines with age, and the strongest decreases have been observed in individuals > 55 years [20]. Normosmia, or normal olfactory function, is assigned to TDI scores higher than 30.3 points; hyposmia, or decreased olfactory capacity, is assigned to TDI scores lower than 30.3; and anosmia, or functional loss of olfactory capacity, is assigned to TDI scores lower than 16.5.

Sniffing Kids test: The “U-Sniff” odor identification test assesses olfactory ability through twelve odors: apple, banana, coffee, cut grass, fish, flower, lemon, onion, orange, peach, and strawberry. These odors are presented using felt-tip pens, with the pen tip positioned approximately 2 cm below the participant’s nostril for 3 s (“U-Sniff,” Burkhart Messtechnik, Wedel, Germany). Each odor is presented separately, and participants identify it by choosing from four descriptors, shown in both text and pictures. The examiner or parents read the descriptors aloud while pointing to the corresponding images. A 20 s pause follows each odor presentation. The total score, ranging from zero to twelve, is based on the number of correct identifications [23].

Eating behavior psychometric tools: A trained dietitian carried out direct interviews with the mothers and their children. Eating behavior was evaluated with 2 different validated psychometric questionnaires: The Child Eating Behavior Questionnaire (CEBQ) and the Food Reinforcement Value Questionnaire (FRVQ). (a) The Child Eating Behavior Questionnaire (CEBQ) is a 35-item questionnaire developed by Wardle et al., 2001 [24], that evaluates eight subscales of eating behavior: Food Responsiveness (FR; 5 items), Enjoyment of Food (EF; 4 items), Emotional Over-Eating (EOE; 4 items), Desire to Drink (DD; 3 items), Slowness in Eating (SE; 4 items), Satiety Responsiveness (SR; 5 items), Food Fussiness (FF; 6 items), and Emotional Under-Eating (EUE; 4 items). Each item is answered on a Likert-type scale with possible scores from 1 to 5, where 1 is complete absence and 5 is the highest intensity of the specific eating behavior The CEBQ, a parent-reported questionnaire, assesses eight distinct traits categorized into two groups: food approach traits (Food Responsiveness, Enjoyment of Food, Emotional Overeating, and Desire to Drink), which indicate a strong appetite and keen interest in food, and food avoidance traits (Satiety Responsiveness, Slowness in Eating, Food Fussiness, and Emotional Under-Eating), reflecting a smaller appetite and lower interest in food. The food ratio was calculated as the quotient of the sum of scores from the “food approach” subscales divided by the sum of scores from the “food avoidant” subscales [24]. (b) Food Reinforcement Value Questionnaire (FRVQ): The FRVQ was developed by Goldfield et al. [25] and further validated by Hill et al. [26]. It is a psychometric tool that evaluates the reinforcement value of food based on the report of children in relation to their feeding behavior. The development of this questionnaire includes 12 items related to the effort that a subject is willing to make to obtain a specific reinforcer (food or a sticker). The choice of the alternatives is offered in a simultaneous schedule, and the preference for food has a higher cost. The task consisted of a 12-item questionnaire designed to evaluate the amount of effort children were willing to expend to obtain a specific reinforcer. At the outset, participants were asked to rank their preferences between a food-based reward (mini cookies) and a non-food reward (stickers). Following this, children expressed their preferences by engaging with a handheld joystick device, which required repeated button presses to access the selected reinforcer. The reinforcement schedule was initiated using an equal fixed-ratio (FR) requirement of 20 responses for both the food and non-food options (e.g., “Would you prefer to press the button 20 times for a cookie or 20 times for a sticker?”).

Subsequent items implemented a progressive fixed-ratio schedule exclusively for the food reward, wherein the response requirement increased in increments of 20 presses per item, culminating in a maximum FR of 240. In contrast, the response requirement for the non-food reward remained constant at 20 presses throughout the task. Upon completion of the 12 items, one item was randomly selected via a draw, and the child was instructed to perform the number of button presses associated with that item in order to receive the corresponding reward. Participants were informed in advance that all earned rewards, both food and non-food, would be delivered at the end of the testing session. The reinforcing value of the food reward was operationally defined as the cumulative number of responses allocated to food-related choices across the task.

Sample size: There have been few studies that have assessed olfactory capacity and obesity in adolescents. Considering that 15% of the general population is hyposmic [27,28,29], the sample size calculated to determine this prevalence is 196 participants. This calculation considers a 95% confidence interval and a power of 80%.

Statistical analysis: All variables were tested for normality using the Shapiro–Wilk test. Descriptive statistics for quantitative variables were reported as means and standard deviations for normally distributed data, or as medians and interquartile ranges for non-normally distributed data. For group comparisons, parametric data were analyzed using the independent samples t-test, whereas non-parametric data were examined using the Mann–Whitney U test or the Kruskal–Wallis test, as appropriate. Associations between variables were assessed using Pearson’s correlation coefficient for parametric data or Spearman’s rank correlation coefficient for non-parametric data.

Multivariate analyses based on multiple linear regression models were employed to examine the relationship between the dependent variable (BMI) and the independent variable (olfactory capacity) while accounting for potential confounding and effect-modifying variables (eating behavior traits) (http://www.stata.com, accessed on 1 January 2023).

## 3. Results

### 3.1. Characteristics of the Participants

A total of 204 participants were included in the study; 48% (*n* = 99) were girls and 51% (*n* = 105) boys. All the variables by gender are presented in Table 1. We found that females had significantly lower height and higher % body fat (*p* < 0.05). In eating behavior traits, girls showed significantly lower scores for Food Responsiveness and Enjoyment of Food and higher Satiety Responsiveness, higher Slowness in Eating, and higher Emotional Under-Eating. Also, girls showed significantly lower global food approach, food ratio, and food choice and higher food avoidance in relation to boys (*p* < 0.05).

When the sample was stratified by nutritional status, there were significant differences in the eating behavior traits FR, EOE, EF, SR, food approach, food ratio, and % food choice (*p* < 0.05) Table 2.

### 3.2. Olfactory Capacity by Nutritional Status

In the global sample, 1.0% of the participants had anosmia, 20.5% had hyposmia, 61.0% had normosmia, and 17.5% were supersmellers. In the total sample, we found that girls had higher % of odor identification (Child Version) in relation to boys (10.1 ± 1.3; 9.5 ± 1.7. *p*-value = 0.01) (Table 1 and Table S1). There was no difference in the olfactory threshold, discrimination, identification, and threshold–discrimination–identification (TDI) score by gender (Table 1) or nutritional status (Table 2) in the total sample. To assess the association between the nutritional condition and olfactory capacity, the variable nutritional status was dichotomized into two groups: normal weight and excess malnutrition (overweight/obesity) (Table 3).

In the total sample, there was no difference in the olfactory threshold, identification, and threshold–discrimination–identification (TDI) score. The discrimination score reached the significance value (12.1± 2.2; 11.4 ± 2.7 *p*-value = 0.05, see Table S3) between the normal weight and excess malnutrition groups. When we stratified the results by nutritional condition and gender, we observed that in girls with obesity, there was a significantly lower odor discrimination capacity (12.4 ± 2.1; 11.5 ± 2.3, respectively, Table S3). In boys, there was no difference in olfactory threshold, discrimination, identification, and threshold–discrimination–identification (TDI) score by nutritional condition.

### 3.3. Anthropometric Variables and Eating Behavior Based on Olfactory Status

We analyzed anthropometric variables and eating behavior based on olfactory status (anosmia, hyposmia, normosmia, and supersmeller), stratified by gender (Table 4). In girls, no significant differences were observed. However, in boys, significant differences were found in total weight (normosmia vs. supersmeller, *p* = 0.02) and specific eating behavior traits, such as Desire to Drink (normosmia: 2.6 (2.0–4.0) vs. supersmeller: 1.6 (1.0–2.6)) and Food Fussiness (hyposmia: 3.16 (2.3–3.8) vs. normosmia: 2.66 (1.83–3.16)). Additionally, anosmic participants had a significantly higher food ratio compared to supersmeller boys, indicating altered eating behavior that is more likely to approach food.

### 3.4. Correlations Between Anthropometric Measurements, Olfaction, and Eating Behavior Traits

Multivariate analyses using multiple linear regression models revealed no statistically significant association between olfactory capacity and obesity after adjusting for potential confounders and effect modifiers.

#### 3.4.1. Normal-Weight Subgroup

A positive association was observed between olfactory identification ability and BMI Z-score (r = 0.23, *p* = 0.04), indicating that participants with better odor identification skills tended to have higher BMI values. Similarly, a positive association was found between the total TDI score, reflecting overall olfactory function, and percentage of body fat (r = 0.24, *p* = 0.03), suggesting that individuals with better global olfactory abilities tended to present higher body fat percentages. Additionally, a lower olfactory threshold, indicating poorer olfactory sensitivity, was significantly associated with higher Emotional Under-Eating (lower food intake in response to negative emotional states such as sadness, anxiety, or stress) (r = −0.34, *p* = 0.002). A significant positive association was identified between odor identification ability and the Emotional Under-Eating dimension of the CEBQ (r = 0.27, *p* = 0.01), indicating that children with better odor identification skills were more prone to reduce their intake when experiencing negative emotions such as sadness, anxiety, or fear.

#### 3.4.2. Overweight/Obesity Subgroup

In the overweight/obesity subgroup, BMI Z-score was positively associated with olfactory threshold (r = 0.18, *p* = 0.04), indicating that higher BMI values were related to poorer olfactory sensitivity. Greater Satiety Responsiveness, defined as feeling satisfied more easily and stopping eating, was positively correlated with TDI score (r = 0.24, *p* = 0.005), suggesting that enhanced olfactory function is linked to improved internal regulation of appetite. Finally, a negative association was observed between olfactory identification ability and the Food Responsiveness dimension of the CEBQ (r = −0.17, *p* = 0.04), indicating that individuals with better odor identification skills were less likely to display reactive eating behaviors in response to external food cues, such as seeing or smelling food, regardless of physiological hunger.

## 4. Discussion

This study evaluated the association between olfactory capacity, body mass index (BMI), body composition, and eating behavior traits in Chilean adolescents aged 10–16 years. In the total sample, 1.0% had anosmia, 20.5% hyposmia, 59.6% normosmia, and 17.6% were classified as supersmellers, consistent with previous reports [30]. The global prevalence of olfactory dysfunction remains uncertain, with estimates indicating that 2–5% of the general population is anosmic and approximately 15% is hyposmic [22,28]. Hyposmic individuals present reduced odor perception, whereas anosmic individuals completely lack olfactory capacity. No significant differences were observed in olfactory performance by gender or nutritional status (normal weight, overweight, or obesity). A recent study evaluating the relationship between olfactory function, eating behavior, and body composition in adolescents aged 10–17 found hyposmia in 21.7% of girls and 39% of boys, with significant differences in the sense of smell between men and women [31].

We hypothesized an inverse relationship between olfactory capacity and BMI; however, no significant associations were detected in the overall sample. A nearly significant trend in odor discrimination emerged, with significant differences by nutritional condition among girls. This results goes in the same direction of the study developed recently by López-Dávalos, who reported decreased retronasal olfactory and taste perception in individuals with obesity, potentially mediated by changes in saliva composition and the oral microbiota, further implicating an oral–brain axis in the perpetuation of obesity [32].

Moreover, the hypothesis that hyposmic individuals would exhibit higher BMI compared to normosmics was not supported. This aligns with the broader literature, where findings on the association between body weight and olfactory function remain inconsistent. Although several studies report reduced olfactory sensitivity in adults with overweight and obesity compared to healthy-weight controls [10,33], consensus regarding the association between BMI and olfactory function remains lacking [2], and some studies have even suggested enhanced olfactory acuity in individuals with obesity [34,35,36]. And at least one study reported no differences in olfactory sensitivity as a function of BMI [14], consistent with our findings in adolescents. Research addressing the relationship between body weight and chemosensory function in children and adolescents is limited. Only one previous study evaluated BMI in relation to olfactory sensitivity and, combined with taste testing, found reduced smell and taste sensitivity in adolescents with obesity (10–16 years) compared to those with normal BMI [11].

We also hypothesized that olfactory status (normosmic vs. hyposmic) would influence eating behavior traits. In line with this expectation, several associations between eating behavior traits and olfactory measures emerged once nutritional status was taken into account. For instance, among normal-weight children, we observed positive associations between odor identification scores and both BMI and percentage of body fat. In addition, an association was found between olfactory threshold (the minimal concentration of an odorant that can be detected) and the ability to regulate food intake under negative emotional contexts. This is consistent with the notion that a lower threshold reflects better integrity and sensitivity of the olfactory system. In children with overweight or obesity, higher BMI was associated with poorer olfactory sensitivity, supporting the notion that excess weight may impair fundamental olfactory processes. Moreover, better overall olfactory performance appeared to relate to greater Satiety Responsiveness, suggesting that enhanced olfactory function may support more adaptive internal appetite regulation. Conversely, children with stronger odor identification abilities showed lower Food Responsiveness, indicating that preserved olfactory skills may help buffer against externally driven eating behaviors.

In this context, one study reported that in children aged 3–9 years, olfactory sensitivity, odor identification, and odor liking were negatively associated with food neophobia [37], highlighting the relevance of olfactory factors in early eating behavior. Additionally, research examining the non-conscious influence of food odors on children’s food choices according to weight status revealed that in normal-weight children, fruity and fatty–sweet odors decreased the likelihood of choosing fruit compared to no-odor conditions, whereas in children with obesity, fruity odors increased fruit selection [38].

Our results may be related to the central role that olfaction plays in food perception and ingestive behavior. Olfactory input contributes up to 80% of flavor perception, meaning that alterations in smell can substantially modify taste experience, food enjoyment, and ultimately dietary choices. In addition, olfaction provides essential protection against environmental hazards such as spoiled food, toxins, and gas [39]. Evidence indicates that hedonic responses to sensory properties of food, particularly smell and taste, modulate eating behavior and may promote overeating and excess weight [40]. Olfactory cues influence appetite regulation, satiety, food selection, and consumption [41].

Therefore, even subtle impairments in olfactory function may influence eating patterns by reducing flavor sensitivity or altering the emotional and motivational responses to food-related cues.

Despite the limited associations detected in our sample, these behavioral patterns highlight the need to examine the physiological pathways through which olfaction and energy balance interact. Olfaction plays a central role in regulating food intake. It is mediated by a two-component molecular system: (i) the signaling component, consisting of odorant molecules perceived as distinct smells largely determined by molecular structure, and (ii) the receptor component, where odor perception corresponds to a unique pattern of neural activity in the receiving organism [33]. Neural activation begins when olfactory sensory neurons (OSNs) detect chemical stimuli via receptor proteins on their surfaces, which recognize compounds by shape [42]. Previous research indicates that appetite-regulating hormones closely linked to olfactory processing play a central role in this relationship [41]. It has also been established that odors guide food search and that eating can modulate olfactory function [35,36,37]. Moreover, exposure to food odors has been shown to increase appetite [38], induce salivation [43], and enhance food-seeking behaviors across species [44].

### Bidirectional Association Between Olfaction and Eating Behavior

Robust evidence supports a bidirectional association between olfaction and eating behavior. Olfactory cues play a critical role in initiating, modulating, and terminating feeding, as well as in food choice and preference. Food odor perception can trigger appetite, facilitate food-seeking, and influence food selection. Transient exposure to food odors generally stimulates appetite and cravings, whereas prolonged exposure may suppress feeding via specific olfactory–hypothalamic circuits [45,46]. The olfactory bulb and related central structures express numerous hormone receptors involved in appetite and metabolism, and olfactory processing is modulated by metabolic states such as fasting, satiety, and overnutrition [45,47].

Neuroimaging and behavioral studies in humans demonstrate that olfactory stimuli can bias food valuation and decision-making, with hunger and satiety states altering both odor perception and neural [48,49]. For example, after eating, individuals are less likely to perceive meal-matched odors as food-dominant, reflecting adaptive changes in olfactory perceptual decision-making that parallel changes in limbic and olfactory brain regions [48]. Cognitive regulation and hormonal status (e.g., ghrelin levels) further modulate the impact of olfactory cues on appetite and food-seeking behaviors [50]. In this sense, Podchinenova et al. (2025) [31] showed that girls with hyposmia showed higher body fat, emotiongenic eating behavior, and correlations between body fat and discriminatory olfactory test results. Boys with hyposmia exhibited positive associations between restrictive eating behavior and BMI, visceral fat, and body fat. The findings suggest that hyposmia is linked to changes in eating behavior and body composition, though the causal direction remains unclear [31].

Emerging evidence underscores the influence of sensory function on dietary behaviors. In a large Italian cohort study, Concas et al. (2022) [50] demonstrated that over 70% of adults presented a reduction in at least one sensory modality, with gustatory impairment being the most prevalent. Notably, diminished sensory capacities were associated with lower food adventurousness and decreased hedonic responses to key dietary components, including vegetables, fish, sweets, and alcoholic beverages. These findings indicate that sensory decline, particularly in older populations, may undermine dietary diversity and adherence to health-promoting dietary patterns [50].

Our findings suggest that olfactory function is associated with the control of eating behavior and body composition, although the direction and significance of these associations appear to vary depending on weight status. Taken together, the results reinforce the idea that individual differences in olfactory sensitivity could play an important role in appetite regulation, emotional response to food, and the development of overweight or obesity.

This study has several limitations and strengths. The fact that the data did not support our hypotheses about olfaction and obesity could be explained in part by some methodological issues. (i) The small sample of our study was obtained based on convenience and cannot be generalized to all Chilean adolescents; (ii) our results could be limited due to the small number of adolescents who met the criteria for hyposmia; (iii) we evaluated eating behavior using psychometric tools. This limitation could result in biases and misinterpretations of our results. Nevertheless, this study has the following strengths: (i) This is the first study in Chilean adolescents that evaluated olfactory capacity and its relation with obesity and eating behavior. (ii) In order to measure eating behavior, we utilized a wide range of tools that were measured face-to-face by highly trained dietitians.

## 5. Conclusions

This study represents the first effort to explore the relationship between olfactory capacity, nutritional status, and eating behaviors in adolescents in Chile. While no direct associations were found between olfaction and weight status in the overall sample, stratified analyses suggest that olfactory differences may influence appetite regulation and specific eating patterns, with variations by sex. These findings highlight the potential modulatory role of the sense of smell in eating behavior, extending beyond its perceptual function, and suggest that its impact on energy balance may be mediated by psychological and behavioral factors. Further research utilizing longitudinal designs and larger, more representative samples will be critical to understanding the causal relationships and underlying biological mechanisms involved.

## Figures and Tables

**Table 1:** Anthropometric measurements in Chilean children.

	All Children (*n* = 204) Median [IQR]	Girls (*n* = 99) Median [IQR]	Boys (*n* = 105) Median [IQR]	*p*-Value
Age (years)	12.0 (11–14)	12.0 (11–14)	12.0 (11–14)	0.57
Weight (kg)	57 (46.1–67.4)	54.9 (46.6–65.7)	58.2 (45.3–68.7)	0.48
height (m)	155 (149–163)	153.5 (149–158.5)	157 (150–168.5)	0.001 *
BMI (kg/m^2^)	22.8 (19.7–26.6)	24.1 (20.1–26.6)	22.41 (19.3–26.5)	0.16
z-score for BMI	1.5 (0.45–2.2)	1.5 (0.65–2.2)	1.3 (0.39–2.2)	0.53
z-score for height	0.14 (−0.4–0.75)	−0.03 (−0.63–0.61)	0.28 (−0.12–0.79)	0.006 *
Waist circumference (cm)	76 (69.2–84.3)	78 (69.5–83)	76 (69–86)	0.83
Waist-to-height ratio	0.49 (0.43–0.54)	0.50 (0.45–0.55)	0.45 (0.43–0.53)	0.18
Body fat mass (%)	28.2 (19.1–36.2)	33.4 (28.1–38.7)	20.6 (14.6–29.4)	0.0001 *
Olfactory capacity				
Odor threshold	8.3 (6.1–11)	8.5 (6.5–11.5)	8.25 (5.75–10.7)	0.28
Odor discrimination	12 (10–14)	12 (10–14)	12 (10–14)	0.51
Odor identification	11 (10–12)	11 (10–13)	11 (10–12)	0.07
TDI score	31.5 (27.5–34.6)	32.5 (29.5–34.5)	30.5 (25.0–34.7)	0.07
Odor identification Child	83.3 (75.0–91.0)	83.3 (75.0–91.0)	83.3 (75.0–91.0)	0.01 *
Eating behavior (CEBQ)				
Food Responsiveness	2.8 (2.2–3.8)	2.6 (2–3.2)	3 (2.2–4.2)	0.008 *
Emotional Overeating	2.25 (1.75–3.2)	2.25 (1.75–3.25)	2.25 (2.0–3.0)	0.95
Enjoyment of Food	3.75 (3.2–4.5)	3.5 (3.0–4.0)	4.25 (3.5–4.7)	0.001 *
Desire to Drink	2.3 (1.3–4.0)	2 (1.33–3.6)	2.3 (1.6–4.0)	0.07
Satiety Responsiveness	2.4 (1.8–3.0)	2.8 (2–3.4)	2.2 (1.6–2.6)	0.001 *
Slowness in Eating	2.2 (1.5–2.8)	2.5 (1.75–3.5)	2 (1.25–2.5)	0.001 *
Emotional Under-Eating	2.25 (2.0–2.8)	2.5 (2–3.25)	2.25 (1.5–3.0)	0.007 *
Food Fussiness	2.83 (2.1–3.6)	3 (2.5–3.6)	2.83 (2.16–3.5)	0.15
Food approach	2.92 (2.35–3.5)	2.78 (2.29–3.20)	3.07 (2.61–3.7)	0.002 *
Food avoidance	2.47 (2.1–2.86)	2.74 (2.27–3.11)	2.29 (1.98–2.64)	0.001 *
Food ratio	116.8 (91.9–157.6)	102.69 (79.4–136.8)	129.9 (106.6–173.7)	0.0001 *
Eating behavior (FRVQ)				
% Food choice	25.0 (8.33–58.3)	16.6 (0–50)	33.3 (8.3–66.6)	0.007

Data are presented as medians and IQRs. * Significant differences between females and males were analyzed with the non-parametric Mann–Whitney test; *p*-values show statistical difference between genders. The data expressed in averages and standard deviation can be found in the Supplementary Materials.

**Table 2:** Anthropometric and eating behavior variables in participants included in the study according to weight status.

	Nutritional Status			*p*-Value
	Normal Weight (*n* = 81) Median [IQR]	Overweight (*n* = 53) Median [IQR]	Obesity (*n* = 71) Median [IQR]	
Age (years)	13 (11–14)	13 (11–15)	12 (10–13)	0.009
Weight (kg)	47.5 (40.9–54.8)	59.4 (47.7–65.5)	67.8 (57.7–78.3)	0.0001
Height (m)	157 (149.15–165.5)	156 (147–162)	153 (149.5–158.9)	0.28
Body mass index (kg/m^2^)	19.22 (18.0–20.6)	23.91 (21.7–25.3)	27.71 (25–30.36)	0.0001
z-score for BMI	0.35 (−0.27–0.65)	1.58 (1.3–1.7)	2.44 (2.19–2.9)	0.0001
Waist-to-height ratio	0.43 (0.41–0.44)	0.5 (0.47–0.52)	0.56 (0.53–0.60)	0.0001
Abdominal circumference (cm)	67.65 (64–72)	78 (73.5–81)	86.5 (81–93)	0.0001
Body fat %	17.7 (13.6–24.0)	28.6 (22.1–35.2)	37.6 (31.7–41.9)	0.0001
Olfactory capacity				
Odor threshold	8.25 (6.2–11.5)	7.25 (5.5–9.5)	8.7 (7.0–11.5)	0.07
Odor discrimination	12 (11–14)	12 (10–14)	12 (9–13)	0.08
Odor identification	11 (10–12	11 (9–12)	11 (10–12)	0.86
TDI score	32 (29–34.7)	31.5 (27–33.7)	31 (27.2–31.7)	0.29
Odor identification Child	83.3 (75.0–91.0)	83.3 (75.0–91.0)	83.3 (75.0–91.0)	0.16
Eating behavior (CEBQ)				
Food Responsiveness	2.8 (2–3.5) ^a^	2.6 (2.0–3.0) ^a^	3.4 (2.4–4.2) ^b^	0.01
Emotional Overeating	2.25 (1.5–2.75) ^a^	2.25 (2.0–2.5) ^a^	3 (2.25–3.5) ^b^	0.001
Enjoyment of Food	3.75 (3–4.25) ^a^	3.75 (3.0–4.25) ^a^	4.25 (3.5–4.75) ^b^	0.003
Desire to Drink	2.33 (1.33–4.0)	2.0 (1.0–3.0)	2.33 (1.66–4.0)	0.13
Satiety Responsiveness	2.5 (2.0–3.0) ^a^	2.6 (2.0–3.0) ^a^	2.2 (1.8–2.6) ^b^	0.005
Slowness in Eating	2.25 (1.75–3.0)	2.0 (1.5–2.75)	2.0 (1.5–2.75)	0.15
Emotional Under-Eating	2.25 (1.75–30)	2.5 (2.0–3.25)	2.5 (2.0–3.0)	0.12
Food Fussiness	2.66 (2.0–3.58)	3.16 (2.66–4.0)	3 (2.16–3.5)	0.08
Food approach	2.81 (2.32–3.18) ^a^	2.67 (2.27–3.17) ^a^	3.22 (2.77–3.84) ^b^	0.0005
Food avoidance	2.48 (2.13–2.82)	2.49 (2.12–3.11)	2.4 (2.04–2.81)	0.47
Food ratio	102.8 (92.8–146.0) ^a^	102.6 (79.3–136.0) ^a^	138.31 (101.8–175.0) ^b^	0.006
Eating behavior (FRVQ)				
% Food choice	41.6 (16.6–62.5) ^a^	16.6 (0–41.6) ^b^	25 (0–50) ^b^	0.005

Data are expressed as medians and IQRs. The data expressed as averages and standard deviations can be found in the Supplementary Materials. Comparisons among the 3 groups were performed using the Kruskal–Wallis test followed by Dunn’s post hoc test for multiple comparisons. Different superscript letters (a, b) within the same row indicate statistically significant differences between groups (*p* < 0.05). CEBQ, Child Eating Behavior Questionnaire.

**Table 3:** Olfactory capacity in adolescents according to nutritional condition.

	Total			Girls			Boys		
	Normal Weight (*n* = 77) Median [IQR]	Excess Malnutrition (*n* = 123) Median [IQR]	*p*-Value	Normal Weight (*n* = 35) Median [IQR]	Excess Malnutrition (*n* = 63) Median [IQR]	*p*-Value	Normal Weight (*n* = 42) Median [IQR]	Excess Malnutrition (*n* = 60) Median [IQR]	*p*-Value
OLFACTORY FUNCTIONS									
Odor threshold	8.25 (6.25–11.5)	8.5 (6–10.5)	0.66	8.5 (7.5–12)	8.5 (6.25–11.5)	0.36	7.5 (6–11)	8.5 (5.75–10.3)	0.91
Odor discrimination	12 (11–14)	12 (10–13)	0.05	13 (11–14) *	12 (10–13) *	0.04	12 (11–14)	12 (9.5–13.5)	0.37
Odor identification	11 (10–12)	11 (10–12)	0.6	11 (10–13)	11 (10–13)	0.81	11 (10–12)	11 (9–12)	0.27
TDI score	32 (29–34.75)	31.2 (27.2–34.5)	0.1	33.2 (30.7–35.7)	32 (28.2–34.2)	0.12	30.6 (28.7–34.2)	20.2 (26.0–34.8)	0.42

Data are expressed as medians and IQRs. The data expressed in averages and standard deviations can be found in the Supplementary Materials. TDI: sum of scores for threshold–discrimination–identification measures. * Significant differences were analyzed with the non-parametric Mann–Whitney test.

**Table 4:** Anthropometric and eating behavior traits by olfactory status.

	Girls					Boys				
	Anosmic (*n* = 1) Median [IQR]	Hyposmic (*n* = 18) Median [IQR]	Normosmic (*n* = 61) Median [IQR]	Supersmeller (*n* = 18) Median [IQR]	*p*-Value	Anosmic (*n* = 1) Median [IQR]	Hyposmic (*n* = 23) Median [IQR]	Normosmic (*n* = 61) Median [IQR]	Supersmeller (*n* = 17) Median [IQR]	*p*-Value
Age (years)	14	12 (11–13)	12 (11–14)	10.5 (10–13)	0.11	13	12 (11–14)	13 (12–14)	10 (10–14)	0.24
Weight (kg)	65.5	57.8 (47.6–68.3)	56.1 (48–65.7)	49.05 (37.8–61.5)	0.28	82.3	60.3 (41.9–69.2)	59.4 (48.2–70.4) ^a^	48.8 (42.2–56.6) ^b^	0.02
Height (m)	160	154.55 (152–158.2)	154 (150–158.9)	146.25 (142.5–156)	0.03	175	157 (150–168.5)	161.6 (152–169.5)	146.4 (143.5–165)	0.08
Body mass index (kg/m^2^)	25.58	24.57 (19.5–27.3)	23.84 (20.6–26.6)	22.13 (19.0–25.8)	0.81	26.8	22.71 (13.3–27.0)	22.78 (20.0–27.1)	21.53 (19.1–22.4)	0.21
z-score for BMI	1.6	1.69 (0.34–2.25)	1.5 (0.66–2.22)	1.54 (0.68–2.41)	0.99	2.32	1.3 (0.31–2.22)	1.44 (0.45–2.23)	0.88 (−0.12–2.03)	0.47
Waist-to-height ratio	0.55	0.50 (0.43–0.52)	0.50 (0.45–0.54)	0.51 (0.44–0.58)	0.69	1.53	0.48 (0.43–0.53)	0.48 (0.43–0.55)	0.45 (0.42–0.51)	0.43
Abdominal circumference (cm)	88	56.9 (69.5–83)	78 (71–83)	75 (68.0–84.2)	0.55	93	75.3 (68–88)	78 (71–87) ^a^	72 (66.5–75.5) ^b^	0.05
Body fat %	25.2	31.8 (26.4–38.7)	34.2 (29.2–38)	32.9 (22.9–41.6)	0.8	27.6	21.9 (14.2–28.9)	21.2 (14.7–32)	17.8 (14.6–26.7)	0.58
Eating Behavior (CEBQ)										
Food Responsiveness	2.8	3 (2.0–4.2)	2.4 (2.0–2.8)	2.7 (2.4–3.6)	0.23	4.4	2.8 (2.0–4.4)	3.2 (2.6–4.4)	2.8 (1.8–3.8)	0.44
Emotional Overeating	1.25	2.5 (1.75–3.75)	2.25 (1.75–3.25)	2.37 (2.0–3.25)	0.41	3.5	2.25 (1.75–3.0)	2.25 (2.0–3.25)	2.25 (1.5–2.5)	0.18
Enjoyment of Food	4	4.0 (3.25–4.5)	3.25 (3.0–3.75)	3.75 (3.5–4.0)	0.08	4.25	4.25 (3.25–4.5)	4.25 (3.5–5.0)	4.25 (3.5–4.5)	0.98
Desire to Drink	1.33	3 (1.66–4.66)	2.0 (1.0–3.0)	2.16 (1.33–3.33)	0.12	4.66	2.33 (1.0–4.0)	2.66 (2.0–4.0) ^a^	1.6 (1.0–2.6) ^b^	0.04
Satiety Responsiveness	2.2	2.3 (1.8–3.6)	2.8 (2.2–3.4)	2.5 (2.0–2.8)	0.25	1.2	2.4 (2.0–2.8)	2.0 (1.6–2.6)	2.2 (1.8–3.2)	0.08
Slowness in Eating	2.25	2.12 (1.5–3.5)	2.5 (1.75–3.5)	2.5 (2.25–2.75)	0.64	1.75	2.0 (1.25–2.5)	1.75 (1.25–2.25)	2.25 (1.75–3.0)	0.26
Emotional Under-Eating	1.75	2.87 (2.0–3.75)	2.5 (2.25–3.25)	2.12 (2.0–2.75)	0.36	2.5	2.5 (2.0–3.0)	2.25 (1.5–3.0)	2.0 (1.5–2.25)	0.23
Food Fussiness	1.66	2.66 (1.83–3.66)	3.16 (2.5–3.83)	2.83 (2.5–3.33)	0.25	1.66	3.16 (2.33–3.83) ^a^	2.66 (1.83–3.16) ^b^	1.33 (2.3–4.0)	0.04
Food approach	2.34	3.15 (2.33–3.89)	2.69 (2.13–3.08)	2.85 (2.4–3.62)	0.08	4.20	2.99 (2.39–3.5)	3.11 (2.72–3.92)	2.72 (2.3–3.2)	0.11
Food avoidance	1.96	2.4 (2.14–3.10)	2.80 (2.4–3.17)	2.58 (2.38–2.78)	0.1	1.77	2.49 (2.07–2.86)	2.21 (1.91–2.49)	2.40 (2–2.7)	0.05
Food ratio	119.2	110.8 (97.6–165.2)	99.26 (70.3–120.7)	111.4 (92.0–139.3)	0.11	236.2 ^a^	128.62 (93.7–157.6)	149.0 (111.67–197.3)	116.01 (94.0–141.2) ^b^	0.03
% Food choice (FRVQ)	83.3	16.6 (0.0–41.6)	16.66 (0.0–41.6)	25 (8.33–50)	0.37	58.3	33.33 (16.6–50.0)	41.6 (8.3–75)	33.33 (0.0–66.6)	0.81

Data are expressed as medians and IQRs. The data expressed in averages and standard deviations can be found in the Supplementary Materials. Comparisons among the four groups were performed using the Kruskal–Wallis test followed by Dunn’s post hoc test for multiple comparisons. Different superscript letters (a, b) within the same row indicate statistically significant differences between groups (*p* < 0.05).

## Data Availability

The data presented in this study are available on request from the corresponding author due to privacy or ethical restrictions.

## Supplementary Materials

The following supporting information can be downloaded at https://www.mdpi.com/article/10.3390/nu17243903/s1, Table S1. Anthropometric measurements in Chilean children. Table S2. Anthropometric and eating behavior variables in the patients included in the study according to the diagnosis. Table S3. Olfactory capacity in adolescents according to nutritional condition. Table S4. Anthropometric and eating behavior traits by olfactory status. Table S5. Anthropometric and eating behavior variables by nutritional status.
