# Supplementary material for: Olfactory Capacity and Obesity in Chilean Adolescents
Source: Nutrients. 2025 Dec 13;17(24):3903. doi: 10.3390/nu17243903 (PMC12736290; doi:10.3390/nu17243903)
Supplement: Supplementary file 1 [file nutrients-17-03903-s001.zip › nutrients-3975031-supplementary.pdf]

nutrients MDPI

# Supplemental Material

**Table S1.** Anthropometric measurements in Chilean children.

| | All Children (n = 204) Mean ± SD | Girls (n = 99) Mean ± SD | Boys (n = 105) Mean ± SD | *p*-Value |
|---|---|---|---|---|
| Age (Years) | 12. 5 ± 1.8 | 12.4 ± 1.8 | 12.5± 1.91 | 0.57 |
| Weight (kg) | 57.6 ± 15.2 | 56.9.6 ± 14.3 | 58.6 ± 16.1 | 0.48 |
| height (m) | 155.9 ± 10.7 | 152.9 ± 7.8 | 158.2 ± 11.9 | 0.001* |
| BMI (kg/m²) | 22.6 ± 4.7 | 24.0 ± 4.7 | 23.1 ± 4.9 | 0.16 |
| z-score for BMI | 1.37 ± 1.2 | 1.41 ± 1.05 | 1.33 ± 1.2 | 0.53 |
| z-score for height | 0.12 ± 0.91 | -0.04 ± 0.97 | 0.28 ± 0.84 | 0.006* |
| Waist circumference (cm) | 76.1 ± 13.4 | 77.0 ± 10.46 | 78.9 ± 14.9 | 0.83 |
| Waist to height ratio | 0.48 ± 0.08 | 0.5 ± 0.07 | 0.5 ± 0.1 | 0.18 |
| Body fat mass (%) | 26.0 ± 10.5 | 32.9 ± 7.8 | 23.1 ± 10.8 | 0.0001* |
| **Olfactory Capacity** | | | | |
| Odor threshold | 8.6 ± 3.5 | 8.8 ± 3.4 | 8.4 ± 3.5 | 0.28 |
| Odor discrimination | 11.7 ± 2.5 | 11.8 ± 2.2 | 11.5 ± 2.7 | 0.51 |
| Odor identification | 10.8 ± 2.1 | 11.1 ± 1.9 | 10.5 ± 2.3 | 0.07 |
| TDI-score | 31.1 ± 5.7 | 31.9 ± 4.9 | 30.4 ± 6.3 | 0.07 |
| Odor identification Child | 9.8 ± 1.6 | 10.1 ± 1.3 | 9.5 ± 1.7 | 0.01 |
| **Eating behaviour (CEBQ)** | | | | |
| Food Responsiveness | 2.9 ± 1.0 | 2.68 ± 0.89 | 3.23 ± 1.1 | 0.008* |
| Emotional Overeating | 2.5 ± 0.92 | 2.5 ± 1.0 | 2.4 ± 0.8 | 0.95 |
| Enjoyment to Food | 3.7 ± 0.82 | 3.5 ± 0.77 | 4.0 ± 0.7 | 0.001* |
| Desire to Drink | 2.5 ± 1.3 | 2.4 ± 1.3 | 2.7 ± 1.3 | 0.07 |
| Satiety Responsiveness | 2.4 ± 0.77 | 2.71 ± 0.82 | 2.1± 0.6 | 0.001* |
| Slowness in Eating | 2.3 ± 0722.9 | 2.59 ± 1.0 | 2.0 ± 0.8 | 0.001* |
| Emotional Under-eating | 2.4 ± 0.89 | 2.63 ± 0.89 | 2.25± 0.8 | 0.007* |
| Food Fussiness | 2.8 ± 0.96 | 2.9 ± 0.93 | 2.7 ± 0.9 | 0.15 |
| Food approach | 2.9 ± 0.76 | 2.79 ± 0.70 | 3.1 ± 0.7 | 0.002* |
| Food avoidance | 2.5 ± 0.6 | 2.72 ± 0.62 | 2.3 ± 0.5 | 0.001* |
| Food ratio | 127.7 ± 55.0 | 110.5 ± 47.7 | 143.9 ± 56.6 | 0.0001* |
| Eating behaviour (FRVQ) | | | | |
| % Food Choice | 37.7 ± 33.2 | 109.0 ± 47.2 | 142.1 ± 54.0 | 0.007 |

Data are presented as mean ± standard deviations. * Significant differences between female and male were analyzed with the nonparametric Mann–Whitney test. *p*-value show statistical difference between gender.

**Table S2.** Anthropometric and eating behaviour variables in patients included in the study according to the diagnosis.

| | Nutritional Status | | | |
|---|---|---|---|---|
| | **Normal Weight (n = 81)** | **Over-Weight (n =53)** | **Obesity (n = 71)** | ***p*-Value** |
| Age (years) | 12.7 ± 1.8 | 12.8 ± 2.0 | 11.9 ± 1.7 | 0.009 |
| Weight (kg) | 48.2 ± 10.2 | 57.3 ± 11.4 | 68.5 ± 15.5 | 0.0001 |
| Height (m) | 157.2 ± 10.7 | 155.2 ± 10.8 | 154.3 ± 9.8 | 0.28 |
| Body Mass Index (kg/m$^2$) | 19.2 ± 1.9 | 23.5 ± 2.1 | 28.4 ± 4.01 | 0.0001 |
| z-score for BMI | 0.19 ± 0.54 | 1.49 ± 0.32 | 2.6 ± 0.56 | 0.0001 |
| Waist to height ratio | 0.43 ± 0.03 | 0.5 ± 0.03 | 0.58 ± 0.07 | 0.0001 |
| Abdominal Circumference (cm) | 68.1 ± 6.0 | 77.4 ± 5.2 | 89.5 ± 13.4 | 0.0001 |
| Body Fat % | 19.4 ± 7.3 | 28.1 ± 7.4 | 37.3 ± 7.2 | 0.0001 |
| **Olfactory Capacity** | | | | |
| Odor threshold | 8.9 ± 3.6 | 7.68 ± 3.4 | 9.0 ± 3.3 | 0.07 |
| Odor discrimination | 12.1 ± 2.2 | 11.6 ± 2.6 | 11.2 ± 2.7 | 0.08 |
| Odor identification | 11.0 ± 2.0 | 10.8 ± 1.9 | 10.6 ± 2.4 | 0.86 |
| TDI-score | 32.1 ± 5.2 | 30.1 ± 5.8 | 30.9 ± 6.0 | 0.29 |
| Odor identification Child | 9.8 ± 1.1 | 9.7 ± 1.6 | 10.0 ± 1.9 | 0.16 |
| **Eating behaviour (CEBQ)** | | | | |
| Food Responsiveness | 2.87 ± 1.0[a] | 2.7 ± 0.97[a] | 3.2 ± 1.08[b] | 0.01 |
| Emotional Overeating | 2.22 ± 0.78[a] | 2.35 ± 0.8 [a] | 2.9 ± 1.0 [b] | 0.001 |
| Enjoyment to Food | 3.69 ± 0.7[a] | 3.6 ± 0.88[a] | 4.0 ± 0.75[b] | 0.003 |
| Desire to Drink | 2.67 ± 1.3 | 2.29 ± 1.3 | 2.7 ± 1.33 | 0.13 |
| Satiety Responsiveness | 2.57 ± 0.7 [a] | 2.57 ± 0.85 [a] | 2.2 ± 0.65[b] | 0.005 |
| Slowness in Eating | 2.43 ± 0.9 | 2.29 ± 1.0 | 2.16 ± 0.88 | 0.15 |
| Emotional Under-eating | 2.29 ± 0.8 | 2.56 ± 0.98 | 2.5 ± 0.88 | 0.12 |
| Food Fussiness | 2.72 ± 0.9 | 3.1 ± 1.0 | 2.86 ± 0.86 | 0.08 |
| Food approach | 2.86 ± 0.6[a] | 2.73 ± 0.72[a] | 3.2 ± 0.79[b] | 0.0005 |
| Food avoidance | 2.5 ± 0.5 | 2.63 ± 0.75 | 2.44 ± 0.52 | 0.47 |
| Food Ratio | 121.3 ± 44.4[a] | 116.6 ± 56.9[a] | 143.2 ± 61.2[b] | 0.006 |
| Eating behaviour (FRVQ) | | | | |
| % Food Choice | 44.0 ± 33.1[a] | 30.5 ± 33.9[b] | 30.1 ± 31.3[b] | 0.005 |

Data are expressed as mean and SD. Comparisons among the 3 groups were performed using Kruskal–Wallis test followed by Dunn's post hoc test for multiple comparisons. Different superscript letters (a, b) within the same row indicate statistically significant differences between groups ($p < 0.05$). CEBQ Child Eating Behaviour.

nutrients

MDPI

**Table S3.** Olfactory capacity in adolescents according to nutritional condition.

| | Total | | | Girls | | |
|---|---|---|---|---|---|---|
| | **Normal Weight (n = 77) Mean ± SD** | **Excess Malnutrition (n= 123) Mean ± SD** | ***p*-Value** | **Normal Weight (n = 35) Mean ± SD** | **Excess Malnutrition (n = 63) Mean ± SD** | ***p*-Value** |
| | | | | OLFACTORY FUNCTIONS | | |
| Odor threshold | 8.8 ± 3.6 | 8.4 ± 3.4 | 0.66 | 9.4 ± 3.2 | 8.5 ± 3.5 | 0.36 |
| Odor discrimination | 12.1 ± 2.2 | 11.4 ± 2.7 | 0.05 | 12.4 ± 2.1* | 11.5 ± 2.3* | 0.04 |
| Odor identification | 11.0 ± 2.0 | 10.7 ± 2.25 | 0.6 | 11.1 ± 2.0 | 11.2 ± 1.8 | 0.81 |
| TDI-score | 32.1 ± 5.2 | 30.6 ± 5.9 | 0.1 | 33.0 ± 4.5 | 31.3 ± 5.1 | 0.12 |

TDI: sum of scores for threshold-discrimination-identification measures; * Significant differences were analysed with the nonparametric Mann-Whitney.

**Table S4.** Anthropometric and eating behaviour traits by olfactory status.

| | Girls | | | | | Boys | | | | |
|---|---|---|---|---|---|---|---|---|---|---|
| | **Anosmia (n = 1) Mean ± SD** | **Hyposmia (n = 18) Mean ± SD** | **Normosmic (n = 61) Mean ± SD** | **Supersmeller (n = 18) Mean ± SD** | ***p*-Value** | **Anosmia (n = 1) Mean ± SD** | **Hyposmia (n = 23) Mean ± SD** | **Normosmic (n = 61) Mean ± SD** | **Supersmeller (n = 17) Mean ± SD** | ***p*-Value** |
| Age (years) | 14.0 | 12.3 ± 1.46 | 12.6 ± 1.88 | 11.5 ± 1.8 | 0.11 | 13.0 | 12.6 ± 1.7 | 12.7 ± 1.8 | 11.7 ± 2.1 | 0.24 |
| Weight (kg) | 65.0 | 56.7 ± 12.7 | 58.3 ± 14.6 | 51.7 ± 14.6 | 0.28 | 82.3 | 57.9 ± 17.7 | 61.5 ± 15.6[a] | 50.2 ± 12.2[b] | 0.02 |
| Height (m) | | | | | | | | | | |
| Body Mass Index ($kg/m^2$) | 25.5 | 23.7 ± 4.1 | 24.4 ± 5.0 | 23.1 ± 4.7 | 0.81 | 26.8 | 22.9 ± 5.1 | 23.8 ± 5.1 | 3.56 ± 15.6 | 0.21 |
| z-score for BMI | 1.6 | 1.37 ± 1.0 | 1.43 ± 1.09 | 1.41 ± 1.0 | 0.99 | 2.32 | 1.21 ± 1.2 | 1.47 ± 1.25 | 1.06 ± 1.25 | 0.47 |
| Waist to height ratio | 0.55 | 0.5 ± 0.06 | 0.5 ± 0.07 | 0.51 ± 0.07 | 0.69 | 0.53 | 0.5 ± 0.08 | 0.51 ± 0.11 | 0.47 ± 0.07 | 0.43 |
| Abdominal Circumference (cm) | 88.0 | 76.1 ± 9.0 | 77.6 ± 10.9 | 75.7 ± 10.4 | 0.55 | 93.0 | 78.7 ± 14.1 | 81.2 ± 16.2[a] | 71.2 ± 8.5[b] | 0.05 |
| Body Fat % | 35.2 | 31.8 ± 8.01 | 33.4 ± 7.7 | 31.9 ± 8.59 | 0.8 | 27.6 | 21.5 ± 8.5 | 24.4 ± 11.7 | 20.5 ± 9.25 | 0.58 |
| **Eating behaviour (CEBQ)** | | | | | | | | | | |
| Food Responsiveness | 2.8 | 3.08 ± 1.1 | 2.51 ± 0.79 | 2.8 ± 0.88 | 0.23 | 4.4 | 3.1 ± 1.1 | 3.32 ± 1.1 | 2.9 ± 1.2 | 0.44 |
| Emotional Overeating | 1.25 | 2.74 ± 1.1 | 2.45 ± 0.93 | 2.68 ± 1.0 | 0.41 | 3.5 | 2.38 ± 0.89 | 2.57 ± 0.85 | 2.17 ± 0.68 | 0.18 |
| Enjoyment to Food | 4.0 | 3.81 ± 0.96 | 3.4 ± 0.74 | 3.76 ± 0.64 | 0.08 | 4.25 | 4.03 ± 0.73 | 4.0 ± 0.85 | 4.0 ± 0.75 | 0.98 |
| Desire to Drink | 1.33 | 3.0 ± 1.5 | 2.2 ± 1.2 | 2.46 ± 1.3 | 0.12 | 4.67 | 2.54 ± 1.41 | 2.92 ± 1.3[a] | 2.0 ± 1.26[b] | 0.04 |
| Satiety Responsiveness | 2.2 | 2.59 ± 0.98 | 2.85 ± 0.81 | 2.46 ± 0.5 | 0.25 | 1.2 | 2.3 ± 0.66 | 2.0 ± 0.61 | 2.45 ± 0.78 | 0.08 |
| Slowness in Eating | 2.2 | 2.39 ± 1.1 | 2.68 ± 1.0 | 2.46 ± 0.84 | 0.64 | 1.75 | 2.0 ± 0.83 | 1.92 ± 0.81 | 2.2 ± 0.77 | 0.26 |
| Emotional Under-eating | 1.75 | 2.8 ± 1.1 | 2.64 ± 0.85 | 2.46 ± 0.76 | 0.36 | 2.5 | 2.45 ± 0.79 | 2.32 ± 0.92 | 1.93 ± 0.7 | 0.23 |
| Food Fussiness | 1.67 | 2.77 ± 1.0 | 3.0 ± 0.95 | 2.88 ± 0.68 | 0.25 | 1.67 | 3.2 ± 0.99[a] | 2.56 ± 0.9[b] | 2.95 ± 1.1 | 0.04 |
| Food approach | 2.35 | 3.1 ± 0.87 | 2.6 ± 0.6 | 2.9 ± 0.68 | 0.08 | 4.2 | 3.0 ± 0.69 | 3.2 ± 0.8 | 2.8 ± 0.72 | 0.11 |
| Food avoidance | 1.97 | 2.6 ± 0.79 | 2.8 ± 0.62 | 2.56 ± 0.4 | 0.1 | 1.78 | 2.5 ± 0.49 | 2.2 ± 0.5 | 2.4 ± 0.49 | 0.05 |
| Food ratio | 119.2 | 135.6 ± 75.5 | 100.7 ± 37.5 | 118.9 ± 36.6 | 0.11 | 236.2[a] | 125.6 ± 38.4 | 155.9 ± 61.8 | 123.2 ± 49.3[b] | 0.03 |
| % Food choice (FRVQ) | 83.3 | 27.3 ± 31.9 | 26.0 ± 27.6 | 36.1 ± 35.2 | 0.37 | 58.3 | 37.3 ± 28.5 | 43.8 ± 36.7 | 38.7 ± 38.8 | 0.81 |

Data are expressed as mean and SD . Comparisons among the four groups were performed using Kruskal–Wallis test followed by Dunn's post hoc test for multiple comparisons. Different superscript letters (a, b) within the same row indicate statistically significant differences between groups ($p < 0.05$).

**Table S5.** Anthropometric and eating behaviour variable by nutritional status.

| | **Girls** | | | | **Boys** | | | |
|---|---|---|---|---|---|---|---|---|
| | **Normal Weight (n = 36) Mean ± SD** | **Overweight (n = 29) Mean ± SD** | **Obesity (n = 34) Mean ± SD** | ***p*-Value** | **Normal Weight (n = 44) Mean ± SD** | **Overweight (n = 24) Mean ± SD** | **Obesity (n = 37) Mean ± SD** | ***p*-Value** |
| Age (years) | 12.5 ± 1.8 | 12.6± 2.0 | 12.0 ± 1.6 | 0.49 | 12.9 ± 1.8 | 13.0 ± 1.9 | 11.7 ± 1.7 | 0.008 |
| Weight (kg) | 45.9 ± 7.7 | 55.9 ± 9.14 | 68.6 ± 14.27 | 0.0001 | 50.2± 11.7 | 59.0 ± 13.8 | 68.4 ± 16.7 | 0.0001 |
| Height (m) | 153.0 ± 8.2 | 152.2 ± 8.0 | 153.5 ± 8.0 | 0.88 | 160.6 ± 11.4 | 158.8 ± 12.7 | 155.0 ± 11.5 | 0.11 |
| Body Mass Index (kg/m$^2$) | 19.4 ± 1.7 | 23.9 ± 2.0 | 28.8 ± 3.8 | 0.0001 | 19.1 ± 2.0 | 22.9 ± 2.3 | 28.0 ± 4.1 | 0.0001 |
| z-score for BMI | 0.2 ± 0.5 | 1.5 ± 0.2 | 2.5 ± 0.4 | 0.0001 | 0.1 ± 0.5 | 1.4 ± 0.3 | 2.6 ± 0.6 | 0.0001 |
| Waist to height ratio | | | | | 0.4 ± 0.02 | 0.4 ± 0.03 | 0.6 ± 0.1 | 0.0001 |
| Abdominal Circumference (cm) | 67.5 ± 5.91 | 77.8 ± 4.6 | 86.4 ± 8.9 | 0.0001 | 68.6 ± 6.2 | 77.0 ± 5.9 | 92.3 ± 16.1 | 0.0001 |
| Body Fat % | 25.6 ± 6.0 | 33.6 ± 4.1 | 39.9 ± 4.6 | 0.0001 | 14.2 ± 3.3 | 21.4 ± 4.4 | 35.0 ± 8.4 | 0.0001 |
| **Eating behaviour (CEBQ)** | | | | | | | | |
| CEBQ | | | | | | | | |
| Food Responsiveness | 2.6 ± 0.7 | 2.4 ± 0.8 | 2.9 ± 1.0 | 0.06 | 3.0 ± 1.2 | 3.0 ± 1.0 | 3.5 ± 1.0 | 0.12 |
| Emotional Overeating | 2.2 ± 0.9 | 2.4 ± 0.8 | 2.8 ± 1.1 | 0.03 | 2.2 ± 0.6 | 2.2 ± 0.6 | 2.9 ± 0.9 | 0.0002 |
| Enjoyment to Food | 3.5 ± 0.7 | 3.2 ± 0.7 | 3.8 ± 0.7 | 0.01 | 3.8 ± 0.8 | 4.0 ± 0.7 | 4.3 ± 0.6 | 0.02 |
| Desire to Drink | 2.5 ± 1.2 | 2.2 ± 1.4 | 2.5 ± 1.2 | 0.33 | 2.8 ± 1.4 | 2.3 ± 1.2 | 2.8 ± 1.4 | 0.40 |
| Satiety Responsiveness | 2.8 ± 0.8 | 2.9 ± 0.7 | 2.3 ± 0.6 | 0.006 | 2.3 ± 0.6 | 2.1 ± 0.7 | 2.0 ± 0.6 | 0.13 |
| Slowness in Eating | 2.7 ± 1.0 | 2.7 ± 1.1 | 2.3 ± 0.9 | 0.22 | 2.1 ± 0.7 | 1.7 ± 0.8 | 2.0 ± 0.8 | 0.03 |
| Emotional Under-eating | 2.4 ± 0.8 | 2.8 ± 0.9 | 2.7 ± 0.9 | 0.13 | 2.2 ± 0.8 | 2.2 ± 0.9 | 2.4 ± 0.8 | 0.48 |
| Food Fussiness | 2.6 ± 0.9 | 3.4 ± 0.8 | 2.86 ± 0.8 | 0.0018 | 2.7 ± 1.0 | 2.6 ± 1.1 | 2.8 ± 0.8 | 0.64 |
| Food approach | 2.7 ± 0.5 | 2.5 ± 0.7 | 3.0 ± 0.8 | 0.04 | 2.9 ± 0.7 | 2.9 ± 0.7 | 3.4 ± 0.7 | 0.011 |
| Food avoidance | 2.6 ± 0.6 | 3.0 ± 0.6 | 2.5 ± 0.5 | 0.043 | 2.3 ± 0.4 | 2.2 ± 0.6 | 2.3 ± 0.4 | 0.21 |
| Food ratio | 107.9 ± 33.5 | 91.7 ± 36.2 | 129.1 ± 61.4 | 0.006 | 132.2 ± 49.4 | 146.7 ± 63.2 | 156.1 ± 58.9 | 0.14 |
| % Food choice (FRVQ) | 37.5 ± 32.4 | 25.2 ± 29.0 | 23.0 ± 27.2 | 0.07 | 49.4 ± 33.1 | 36.8 ± 38.7 | 36.7 ± 33.7 | 0.13 |

Data are expressed as mean and SD. Comparisons among the four groups were performed using Kruskal–Wallis test followed by Dunn's post hoc test for multiple comparisons.
